# From Innate Immunity to Cancer Therapy: Antimicrobial Peptides as Emerging Anticancer Agents

**DOI:** 10.3390/ijms27125179

**Published:** 2026-06-08

**Authors:** Neha Raut, Saeed Vohra, Pooja Kaushalye, Sainath Mane, Divya Malode, Milind Umekar, Abdulrahman Mohammed Alhudhaibi, Anis Ahmad Chaudhary, Rashmi Trivedi

**Affiliations:** 1 Department of Quality Assurance, Smt. Kishoritai Bhoyar College of Pharmacy, Kamptee, Nagpur 441002, Indiakaushalyepooja@gmail.com (P.K.);; 2 Department of Anatomy and Physiology, College of Medicine, Imam Mohammad Ibn Saud Islamic University (IMSIU), Riyadh 11623, Saudi Arabia; 3 Department of Pharmaceutics, Smt. Kishoritai Bhoyar College of Pharmacy, Nagpur 441002, India; 4 Department of Biology, College of Science, Imam Mohammad Ibn Saud Islamic University (IMSIU), Riyadh 11623, Saudi Arabia

**Keywords:** antimicrobial peptides, anticancer peptides, cancer therapy, immunogenic cell death, nanocarrier delivery

## Abstract

The potential for the use of antimicrobial peptides (AMPs) as anticancer agents has garnered much interest because of their selective cytotoxicity to tumor cells and ability to evade multidrug resistance mechanisms. AMPs are shorter cationic amphiphilic molecules, part of our innate immune system, with direct membrane-disruptive activity and immunomodulatory effects. Anticancer peptides (ACPs) can be derived from natural biophysical sources or synthetically engineered, taking advantage of the unique biophysical properties of cancer cell membranes to exert their anti-tumor activities rapidly and often without significant effects on normal tissues. Advances in peptide engineering, such as D-amino acid substitution, cyclization, and PEGylation, combined with nanocarrier systems, have provided opportunities to improve peptide stability, bioavailability, and delivery to targeted sites. Studies in preclinical and clinical models show promise, indicating that AMPs and ACPs can induce immunogenic cell death, modify tumor microenvironments, and be used in combination with more conventional therapies. While the promise of AMPs and ACPs as relatively novel cancer therapeutics is substantial, challenges such as proteolytic degradation, dose-dependent toxicity, costs for production, and regulatory hurdles are notable. This review organizes the current literature on classification, mechanism(s) of action, delivery strategies, preclinical and clinical data, and provides areas for future work to improve and help speed their clinical translation as new cancer therapies.

## 1. Introduction

Cancer is increasingly becoming a severe public health problem and is expected to impose an enormous and massive strain on the world. In 2022, cancer was reported in a projected 20 million people globally, and 9.7 million cancer deaths were recorded in the same year. The number of new cancer instances per year is expected to grow to more than 35 million by 2050 because of an increase in population, aging, and lifestyle-related cancer risks [1]. The reasons behind this trend include aging populations, changes in lifestyles, and the presence of cancers related to persistent infection, especially in resource-poor countries. Strategies for avoidance are essential to counteract this problem [1].

Many aspects influence cancer development, including infections, genetics, dietary routines, hormonal imbalances, and immune status. However, traditional treatments (surgical interventions, chemotherapy, and radiation therapy) are not specific enough, hence influencing healthy tissue along with cancerous cells, leading to toxicity and impaired quality of life. Therefore, particular treatments are required for future developments [2]. T&CM can help cancer patients through enhanced and upgraded well-being and treatment side-effect management [3]. AMPs are essential and pivotal components of the innate immune system, forming the primary defense against invading germs in a multitude of species, including bacteria and humans [4]. AMPs are molecules formed by fewer than 100 amino acids that are encoded by genes, acting as broad-spectrum antimicrobials against bacteria, fungi, and even viruses and forming vital components of innate immunity, offering immediate defense against microorganisms. As mentioned, “AMPs are a rudimentary innate immune mechanism existing in all living beings, offering the primary defense line against invading germs” [5].

In broad and inclusive terms, AMPs are positively charged, with a total charge of 3 +ions. AMPs may moreover be referred to as host defense peptides or innate defense regulatory peptides, which function nonspecifically by targeting microbial components involved in the induction of the immune system [6]. AMPs are found thoroughly in nature, present in bacteria, insects, amphibians, fish, birds, and mammals, including humans. Enhancing the presence of AMPs in nature, “AMPs are an essential component of defense processes present in all forms of life, and their presence across the whole and complete evolutionary spectrum proves their effectiveness and significance in restricting the impact of germs” [7].

They furthermore exhibit a relatively low potential for building bacterial opposition since they are speedy in action, potent, and wide-spectrum in their antimicrobial action towards a huge variety of microorganisms, covering from multidrug-resistant Gram-negative and Gram-positive bacteria to parasites, fungi, and viruses. This unique set of characteristics makes AMPs promising options to conventional antibiotics [8].

Apart from their antimicrobial actions, AMPs exhibit various other biological functions, including immunomodulatory effects, antiviral effects, antitumor effects, and wound healing attributes. It is noted that “AMPs, separately from their microbicidal action in host tissues, consequently display a total and whole range of activities incorporating cell proliferation, stimulation of the immune response, and cytotoxicity towards tumor cells” [5,9]. While microbial lytic peptides and anticancer peptides have comparable physico-chemical characteristics, antimicrobial peptides and anticancer peptides are not identical. AMPs are a broad category of host-defense peptides, showing antimicrobial activity primarily against bacteria, fungi, viruses, and parasites, and the antimicrobial anticancer peptides (ACPs) represent peptides that are being tested or designed with the chief function of a distinct anticancer activity. Most crucially, it should be noted here that some AMPs may have anticancer attributes, but not all. In the setting of this review, the term “ACPs” is reserved for peptides that have an anticancer mechanism or therapeutic effect [10].

The varied and distinct capabilities displayed above illustrate their role as multifunctional effectors of the innate immunity system. Antimicrobial peptides are referred to as amphipathic and cationic peptides that have an uncommon mechanism of action. Upon encountering the cell membranes of germs, the peptides will adopt an amphipathic conformation, which permits them to form pores leading to membrane interruption and eventually resulting in cell demise [8]. Membrane targeting capability against a broad array of microbial germs and non-pathogens is necessary and fundamental for the effectiveness of AMPs in their antimicrobial activity. The following are among the qualities that make AMPs fitting for innate defense functions. First, AMPs possess robust microbicidal activity at concentrations as low as micromolar. Second, AMPs cause bacteria to die swiftly once exposed to AMPs. Lastly, there is no evidence to suggest that AMPs support antimicrobial opposition. The mechanism through which AMPs act against bacteria requires more than disrupting cell membrane stability [10]. Such attributes allow these molecules to exhibit antimicrobial effects towards various and several resistant strains of bacteria, comprising, but not limited to, multidrug-resistant methicillin-resistant Staphylococcus aureus, Clostridium difficile, Streptococcus pneumoniae, and Enterococci [11]. Typically, AMPs can be described as peptides that show conservation, amphipathy, and serve as the primary obstacle against any disease-causing microorganisms. These peptides act by virtue of electrostatic interactions between their structures and microbial membranes or intracellular components, thereby producing membrane permeabilization and/or interruption [6,12,13].

## 2. Classification and Sources of AMPs

Antimicrobial peptides comprise a heterogeneous group of molecules designated by both structure and net charge, which correlate with biological activity and selectivity. Bioactive peptides could be grouped into a special class of ACPs, which can be natural peptides or synthetically synthesized for anticancer activities. The biological goals and therapeutic applications of the ACPs are occasionally equivalent to those of the AMPs, yet they show structural features that are distinct [14,15,16].

### 2.1. Structural Classification

AMPs and ACPs are classified according to their structural features, as shown in Table 1. These features are responsible for determining their stability, membrane association, and particular action against cancerous cells.

### 2.2. Charge-Based Classification

AMPs and ACPs can be broadly categorized according to their charge, a key factor in their selective recognition of microbial and cancer cell membranes. Most AMPs are positively charged, and they interact strongly with negatively charged microbial cell membranes, mainly as a result of the presence of anionic phospholipids (phosphatidylserine and phosphatidylglycerol), which impart a net negative surface charge [20]. On the other hand, mammalian cell membranes have an asymmetric distribution of phospholipids with most anionic lipids found in the inner leaflet, which renders the outer surface of the membrane close to neutral and thus contributes to the selective killing of microbial cells by cationic peptides [21]. These positively charged peptides commonly use mechanisms, such as membrane permeabilization, depolarization, leakage, and ultimately, cytoplasm leakage and cell lysis, to kill microorganisms [22]. A subset, known as linear cationic antimicrobial peptides (LCAPs), possesses amphiphilicity and flexibility, and can exert both membrane-disruptive and intracellular mechanisms [23]. Likewise, in cancer treatment, cationic ACPs specifically target cancer cells because cancer cell membranes are more negatively charged, attributed to the presence of glycoproteins, glycolipids, proteoglycans, and so on. These ACPs employ the carpet model, barrel-stave model, and toroidal pore model to disrupt the membrane and mediate cell death. Therefore, a high net positive charge is crucial for improving anticancer activity and selectivity. By contrast, anionic antimicrobial peptides (AAMPs) and anionic ACPs are understudied, typically with a net charge between −1 and −7 and a length from 5 to about 70 amino acids; these peptides may present different cofactor-dependent mechanisms and new approaches for characterization and development as therapeutic agents [22,23,24].

### 2.3. Source Diversity

The diversity in the sources of antimicrobial peptides and anticancer peptides is a consequence of their ubiquitous presence across different biological kingdoms, and of the progress in synthetic and engineered systems. This source diversity impacts their structure, function, and applications. Plant, animal, and microbe-derived AMPs and ACPs have distinct modes of action, including membrane disruption, apoptosis initiation, and immune stimulation, whereas engineered and synthetic AMPs and ACPs feature enhanced stability, specificity, and bioavailability. Table 2 below illustrates the primary sources of AMPs and ACPs, some of their examples, and the main key features.

## 3. Mechanisms of Anticancer Action

**Figure 1:**
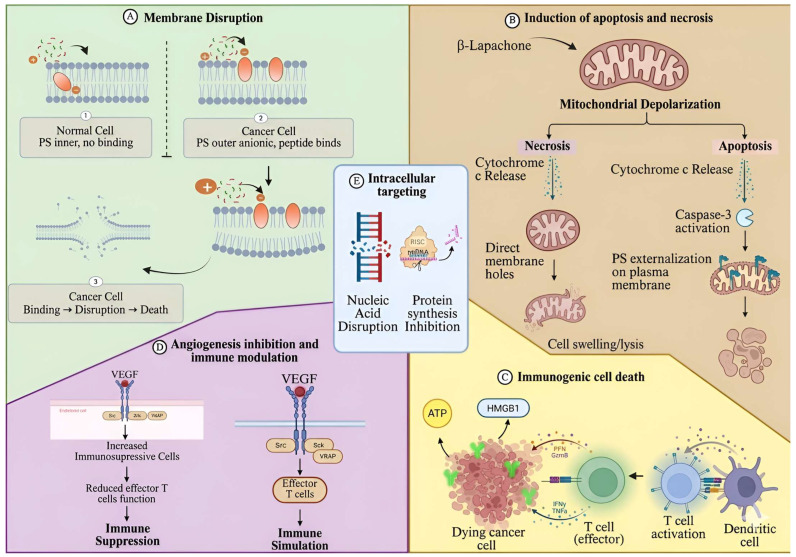
Schematic representation of the multifaceted mechanisms of anticancer action of antimicrobial peptides, including (**A**) membrane disruption, (**B**) induction of apoptosis and necrosis, (**C**) immunogenic cell death, (**D**) angiogenesis inhibition and immune modulation, and (**E**) intracellular targeting.

Anticancer peptides exert their antitumor effects through multiple, often overlapping mechanisms that collectively contribute to selective tumor cell killing while sparing normal cells. These mechanisms include direct membrane disruption, induction of apoptosis and necrosis, immunogenic cell death, inhibition of angiogenesis with immune modulation, and intracellular targeting of vital biomolecular processes. The range of these anticancer activities is schematically displayed in Figure 1.

### 3.1. Membrane Disruption as a Primary Mechanism of Anticancer Action

Cells and biomembranes have electrical potential and fluidity. Obviously, an increase in the fluidity of the membrane is a fundamental element of the activity of the ACP. The fluidity of cancer cell membranes is moreover greater than that of normal cells, which destabilizes normal cell membranes, assisting in increasing the lytic activity of ACPs. In addition, the net negative charge of cancer cell membranes arising from the increased expression of anionic molecules furthermore offers crucial and vital electrostatic interactions with cationic ACPs, which are significant and essential contributors to their selectivity. The intricacy of the composition of lipids and cholesterol. Complexity of lipids and cholesterol. The discussion below of the importance of the modified lipid composition refers to model membranes found in the pure lipids PC, PC/PG and PC/PS, but the actual biomembranes contain “immense variety of lipids, each possessing a variety of physicochemical characteristics. As far as the presence of cholesterol, the inquiry admits that it can have a ”marked effect on ACP behavior“ but its concentration is extremely dissimilar in distinct cell sorts and occasionally it can even be absent in some. Someone whose controls are diverse and heterogeneous is referred to as possessing ‘heterogeneity and multifactorial selectivity’. The study highlights that there is more to being selectively reactive than just simply replying to PS exposure. Spatial distribution: The spatial distribution of charged moieties of the lipid headgroups and along the peptide sequence is fine-tuned by binding. Intra-molecular flooding of peptides by means of water molecules of different binding homologies.

However, for the peptides, the three models suggested therein revealed three distinct binding modes– an extremely helical “carpet” model (A), a partly helical surface binding (B) and a perpendicular “antenna-like” model (C)—suggesting that each peptide may bind to a dissimilar feature in the binding surface and, therefore, that differences in the binding modes may reflect differences in the binding preferences of the peptides. In addition, the authors chose to employ red blood cell-derived extracellular vesicles (REVs), which have a more physiological lipid and associated protein composition, instead of straightforward lipid models. Such interactions correlate with the spectroscopy and molecular dynamics simulations, and the authors conclude that ACP selectivity is undoubtedly a high-intricacy phenomenon in which the minimal differences in sequence and charge distribution of the peptide with that of the complicated surface of a tumor cell membrane play a role [31].

Selective discrimination between cancerous and healthy mammalian cells is not absolute; nevertheless, with greater concentrations of multiple ACPs, it may furthermore help with the above cancerous discrimination, consequently assisting in hemolytic activity and producing systemic toxicity [32].

The presence of PS on the cancer cell surface endows the overall anionic character to the membrane, which results in a distinct and individual binding of cationic peptides and consequent destabilization of the membrane, culminating in the demise of tumor cells. The effect process places membrane interruption as a chief and speedy anticancer response (Figure 1A) [33]. One such mechanism is the LTX-315, which selectively and irreversibly engages with PS-rich anionic cancer cell membranes in an electrostatic manner to disrupt the membrane and cause cell demise [34]. PPS1D1, like other cancer therapies, creates anticancer effects through electrostatic interactions with cell surfaces that are rich in phosphatidylserine and have atypical and atypical glycosylation. The result is a destabilization of the membranes, permeability of the membranes, and the precise devastation of tumor cells, supporting the conclusion that the membrane is disrupted by peptides in order to generate anticancer effects, which is a function of peptide-mediated anticancer activity (Figure 1A) [35].

### 3.2. Induction of Apoptosis and Necrosis

The mode of action of anticancer peptides can extend past the direct damage of a cell membrane; they can furthermore invoke programmed and/or non-programmed cell death pathways by depolarizing mitochondria and activating caspases as a first regulatory incident. For instance, β-lapachone’s capability to induce apoptosis in human ovarian, colon, and lung cancer cells is supported by, amongst other things, the externalization of phosphatidylserine, the abundant presence of sub-G1 cells, and activation of caspase-3 [36]. In comparison, the compound causes necrosis in various breast cancer cell lines (MCF-7, 21 MT, 21 PT, and 21 NT), with no evidence of caspase-3 activation or classical apoptosis markers, whereas apoptosis and necrosis both occurred before there were swift and speedy mitochondrial cytochrome c release and depolarization of the mitochondrial membrane, demonstrating mitochondrial dysfunction to be a central and vital factor that determines cell fate (Figure 1B) [37]. In conclusion, β-Lapachone can result in either apoptosis or necrosis, depending upon the type of cancer cell that it is targeting, through operations involving mitochondrial membrane depolarization and cytochrome c release [38]. The mitochondria regulate both caspase-dependent and independent apoptotic pathways, producing the release of apoptogenic (apoptosis-inducing) aspects and the first and beginning loss of mitochondrial membrane potential (ΔΨm) as a result of cellular stress-mediated mitochondrial outer membrane permeabilization and activation of the mitochondrial permeability transition pore (MPTP) (Figure 1B) [39]. While apoptosis, accomplished by anti-CD95 due to cytochrome c release and substrate cleavage, occurs through caspases in HepG2, menadione induces necrosis through cytochrome c release, but does not activate caspase apoptosis; instead, membrane rupture occurs quickly, and there is no indication of nuclear condensation. These data highlight the different systems invoked by peptide-mediated cell demise [40]. 

### 3.3. Immunogenic Cell Death (ICD)

Danger-associated molecular patterns are released by dying cells and can contain ATP, Calreticulin on the cell surface, and HMGB1 [41]. By stimulating antigen-presenting cells (via DAMPs) and priming cytotoxic T-cells, ICD elicits a response from the innate immune system as well as an adaptive immune system to support effective total and whole integration of peptide-based immunotherapies with ICD-induced DAMP generation (Figure 1C) [42].

### 3.4. Angiogenesis Inhibition and Immune Modulation

In addition to direct cytotoxic mechanisms, anticancer peptides can modulate the tumor microenvironment by targeting angiogenesis and immune suppression. Vascular endothelial growth factor (VEGF)-targeted anti-angiogenic therapies have been shown to reshape antitumor immunity by reducing regulatory T cells and myeloid-derived suppressor cells within tumors and peripheral circulation (Figure 1D) [43]. VEGF signaling suppresses immune responses by inhibiting T-cell activity and promoting immunosuppressive cellular populations. Conversely, blockade of the VEGF/VEGFR signaling axis restores immune competence by enhancing effector T-cell infiltration, increasing cytokine production, and reducing immunosuppressive cell populations. This dual anti-angiogenic and immunomodulatory effect underscores the importance of VEGF-targeting strategies in peptide-based anticancer therapy [44].

### 3.5. Intracellular Targeting

Beyond membrane interruption, multiple antimicrobial peptides penetrate cancer or microbial cells and interfere with necessary and fundamental intracellular processes. Peptides such as buforin II, indolicidin, and microcin B17 inhibit nucleic acid metabolism by binding to DNA or RNA, thereby blocking replication or transcription. Buforin II preferentially binds to DNA while indolicidin interacts with apurinic DNA sites to inhibit nucleic acid synthesis. Other AMPs have been found to inhibit protein biosynthesis by targeting ribosomes or by disrupting chaperone-assisted protein folding. Proline-rich peptides have been displayed to selectively inhibit the DnaK chaperone system to induce intracellular killing (Figure 1E) [45].

## 4. Structure-Activity Relationship (SAR)

### 4.1. Roles of Net Charge, Hydrophobicity, Amphipathicity, and Peptide Length

Understanding the SAR of antimicrobial peptides and anticancer peptides is crucial for optimizing their selectivity, potency, and safety, as shown in Table 3 [46].

### 4.2. Rational Design Approaches and Synthetic Modifications

**Figure 2:**
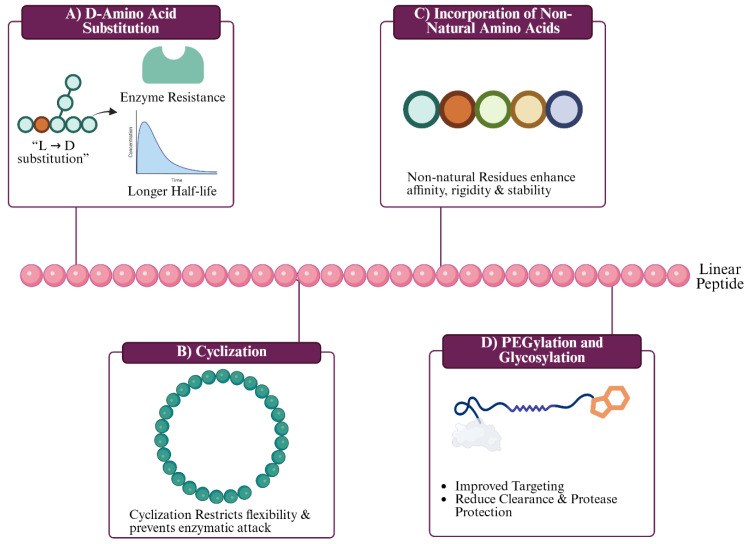
Rational design approaches for improving peptide stability and pharmacokinetics: (**A**) D-amino acid substitution enhances resistance to enzymatic degradation, (**B**) Cyclization restricts conformational flexibility, improving stability and bioactivity, (**C**) Incorporation of non-natural amino acids increases receptor affinity and metabolic stability, (**D**) PEGylation and glycosylation shield peptides from proteolysis, reduce renal clearance, and enhance in vivo performance.

Common strategies such as D-amino acid substitution, cyclization, PEGylation, and incorporation of non-natural amino acids help overcome challenges like rapid enzymatic degradation, limited systemic half-life, and poor bioavailability [57], as shown in Figure 2.

**(A)**  **D-Amino Acid Substitution**

Incorporating D-amino acids is one of the most effective approaches to enhance peptide stability. Incorporation of D-amino acids into biologically active peptides can improve metabolic stability compared with L-amino acids, given that few human enzymes hydrolyze peptide bonds with D-amino acids [58]. A case of a timeless case is desmopressin, in which the addition of D-arginine enhances the pharmacokinetics and metabolic stability. In the identical way, D-Phe-containing analogs of somatostatin, octreotide, exhibit improved enzymatic opposition and a longer human half-life of almost 2 h [59].

**(B)**  **Cyclization**

Another widespread approach that has been used to increase the biological activity and longevity of peptides is cyclization. Limiting conformational versatility permits cyclic peptides to be substantially resistant to enzyme decline. Cyclization is commonly utilized to increase conformational stability and/or biological activity over linear analogs. The arising molecules are not easily broken down by peptidases due to the constraint of conformations and/or the lack of amino and carboxyl ends [60].

**(C)**  **Incorporation of Non-Natural Amino Acids**

In addition to the application of D-amino acids, the incorporation of other non-natural amino acids is a well-planned approach to enhancing the design of peptides. The changes play a role in enhancing receptor affinity, structural rigidity, and metabolic stability. As mentioned, cyclization and non-natural amino acid introduction are vital and crucial in the stabilization of peptide ligands, which are used in therapeutic and imaging applications [61].

**(D)**  **PEGylation and Glycosylation**

PEGylation and glycosylation are other chemical changes that have an immense effect on the pharmacokinetic behavior of peptides. The plans improve renal clearance, which is reduced by growing molecular size and securing the peptide backbone by inhibiting proteolytic enzymes. PEGylated bombesin analogs, which exhibited considerably better in vitro and in vivo stability, and moreover enhanced tumor absorption [59]. PEG-conjugated GIRLRG peptide, which was displayed to have better metabolic stability and specificity to GRP78-overexpressing tumor cells [62].

### 4.3. Computational Prediction and Peptide Design Tools for Amps and Acps Discovery

A broad repertoire of computational strategies has been developed to aid the prediction, design, and optimization of Amps and Acps. These techniques are established on sequence analysis, machine learning, structural simplification, and pattern recognition to determine peptides of increased activity and reduced toxicity [63].

**(A)**  **Direct Sequence Analysis**

Initial background Raghava and others proposed three prediction systems employing the basis on the preferences of AMPs sequences in the Antimicrobial Peptide Database (APD): (1) Support Vector Machine (SVM), (2) Artificial Neural Network (ANN), (3) Quantitative Matrix (QME). Among these, the SVM model, which used both N- and C-terminal sequences, had an almost predictive accuracy of 99% in identifying AMPs [64].

**(B)**  **QSAR-Based Supervised Learning Techniques**

PCA with QSAR descriptors has found exhaustive application in modeling the antibacterial activity with a prediction accuracy of up to 97%. Monitored ML systems like SVMs and ANNs are capable of anticipating the AMP actions in opposition to Pseudomonas aeruginosa and measuring the power of antimicrobials and the level of hemolytic toxicity, consequently facilitating optimization of safer and more effective peptides [65].

**(C)**  **Linguistic Models and Reduced Amino Acid Alphabets**

Other techniques for AMP design: Pattern recognition and grammar-based models provide alternative methods to AMP design. Teiresias, a pattern discovery algorithm, has been applied to extract regular grammar out of APD sequences and used to generate new AMPs rationally. Small amino acid alphabets, like Protein Blocks, are helpful and beneficial in identifying significant structural motifs, and have been used to define families and subfamilies of defensins. By simplifying sequence space without losing biologically relevant information, these instruments support productive and effective peptide classification and design [66].

**(D)**  **CAMPR3**

CAMPR3 is a database and calculator of antimicrobial peptides that is built on machine-learning methods, including SVM, random forest, and ANN, to support the design and identification of novel AMPs in silico [67].

**(E)**  **AMPs Scanner v2**

The AMPs Scanner v2 uses a framework that is based on convolutional neural networks that learns discriminative peptide sequence features directly, without the need to engineer features manually [68].

**(F)**  **AntiCP 2.0**

AntiCP 2.0 is a machine-learning-based anticancer peptide predict-and-design code that uses primary sequence to predict and design anticancer peptide sequences, which may be used in in silico to prioritize peptides with potential anticancer applications [69].

**(G)**  **Rosetta Peptide Design**

The rational design of peptides can be performed using Rosetta, which consists of flexible backbone sampling coupled with sequence optimization using an all-atom energy functional and can be used to systematically find peptide sequences and conformation states with increased stability and affinity to targets [70].

**(H)**  **Other Computational Approaches**

Weighted finite-state transducers, designed to classify 30-mer peptides as AMPs. Combined methods based on sequence alignment, feature extraction, and feature selection take advantage of both evolutionary and discriminative information to enhance prediction accuracy. All these complementary tools are a part of strong identification and optimization of AMP candidates [71].

## 5. Delivery Strategies for ACPs in Cancer Therapy

### 5.1. Nanocarrier Delivery

**Figure 3:**
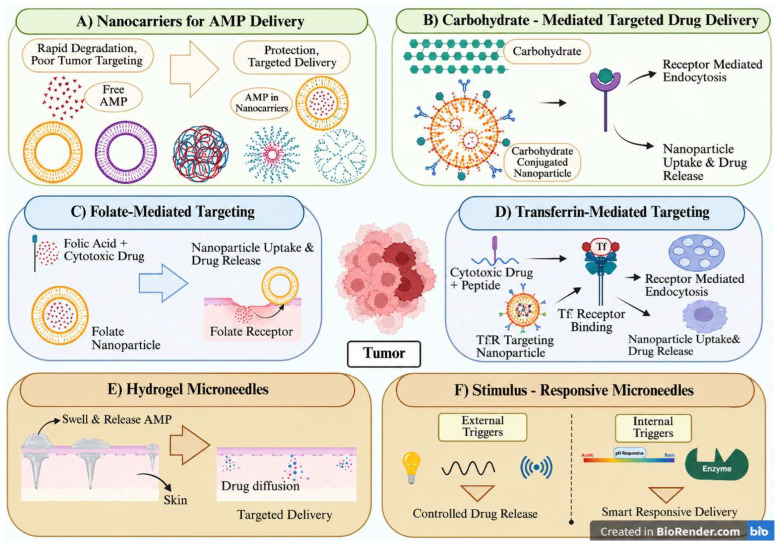
Advanced strategies for targeted delivery of AMPs and anticancer therapeutics: (**A**) Nanocarrier-based delivery systems enhancing AMPs stability, protection from enzymatic degradation, and tumor targeting. (**B**) Carbohydrate-mediated targeted drug delivery via receptor-mediated endocytosis. (**C**) Folate-mediated nanoparticle targeting exploiting overexpressed folate receptors on cancer cells. (**D**) Transferrin-mediated targeting through transferrin receptor (TfR)-dependent endocytosis. (**E**) Hydrogel microneedles enabling localized and controlled AMPs delivery through skin penetration. (**F**) Stimuli-responsive microneedle systems providing controlled and smart drug release in response to internal or external triggers.

Nanotechnology delivery systems have become potent to enhance the therapeutic effectiveness of AMPs in the treatment of cancer. These approaches help to improve peptide stability, bioavailability, tumor penetration, and controlled release, and reduce systemic toxicity. A number of nanocarrier systems are being used or being examined, such as liposomes, niosomes, polymeric nanoparticles, polymeric micelles, and dendrimers, to be used as AMPs to cure cancer [72], as shown in Figure 3.

**(A)**  **Niosomes**

Niosomes are a type of vesicle that are non-ionic surfactant-based vesicles and whose application as a carrier of AMPs has been growing in appeal because they are extremely stable structurally, biocompatible, and have a high drug encapsulation capability. Niosomes are structurally bilayer vesicles chiefly made up of non-ionic surfactants (for example, Spans and Tweens), cholesterol, and, in specific formulations, charged additives like diacetyl phosphate to enhance stability and surface characteristics of the vesicles. The bilayer structure permits encasing hydrophilic (in the aqueous core) and lipophilic (in the bilayer membrane) agents [73]. Niosomes have a number of benefits over traditional phospholipid-based liposomes, such as improved chemical and physical stability, decreased immunogenicity, increased shelf life, biodegradability, low toxicity, and low cost of production in large and substantial quantities [72,73,74]. These attributes render niosomes notably suitable and proper to deliver labile biomolecules like AMPs. The lipid-based systems come with a variety of classes and differ substantially in their drug loading capability, release profile, and stability, such as small and huge and massive unilamellar vesicles (SUV and LUV), giant unilamellar vesicles, disomas, aspasomes, proniosomes, and polyhedral niosomes [75]. Typical procedures of preparation are thin-film hydration, reverse-phase evaporation, ether injection, and microfluidization [76]. Niosomes have been examined in conditions of delivery of small molecules, natural products, proteins, genes, and vaccines. They are promising vectors of AMPs in the treatment of cancer due to their capability to improve tumor targeting [72].

**(B)**  **Liposomes**

Liposomes are spherical vesicles formed of phospholipid bilayers and are among the most recognized nanocarriers in drug delivery. Their biocompatibility, structural similarity to biological membranes, and capability to encapsulate both hydrophilic and hydrophobic drugs make them appealing platforms for AMPs delivery. Liposomal encapsulation improves peptide stability, prolongs circulation time, and enhances tumor collection via the improved permeability and retention (EPR) effect. Characterization of liposomes usually requires techniques such as dynamic light scattering (DLS) for size distribution, zeta potential analysis for surface charge, and Förster resonance energy transfer (FRET) assays to evaluate membrane integrity and stability under physiological conditions [77].

**(C)**  **Polymeric Nanoparticles**

Polymeric nanoparticles are versatile AMP carriers as a result of their capability to be customized in conditions of their physicochemical attributes. The chief traits are the capability to load small molecules, peptides, proteins, or nucleic acids, size, morphology, and surface chemistry can be customized, sustained, and controlled release. Characterization techniques such as atomic force microscopy (AFM) and differential scanning calorimetry (DSC) assist in the evaluation of polymer-drug interactions, morphology, and loading productivity [77,78].

**(D)**  **Polymeric Micelles**

Polymeric micelles self-assemble from amphiphilic block copolymers into a core–shell nanostructure, where the hydrophobic core solubilizes lipophilic drugs and the hydrophilic shell prolongs systemic circulation; this architecture enhances the delivery of poorly soluble AMPs and strengthens tumor accumulation [79].

**(E)**  **Dendrimers**

Dendrimers are highly branched, monodisperse, and have many functional groups available on the surface to allow for precise drug loading, tailorable surface interactions, and controlled drug-release kinetics. Dendrimers have a multivalent structure that allows for stratified targeting and delivery of AMPs in the treatment of cancer [78].

### 5.2. Role of Nanocarriers in AMP Delivery

Nanocarriers such as niosomes, liposomes, polymeric nanoparticles, micelles, and dendrimers are able to improve the safety, tumor targeting capacity, and therapeutic effect of AMPs. Formulations based on the nanotechnology platform are becoming a promising approach to improve the limitations of conventional cancer treatments, due to prolonged circulation time, protection from enzymatic degradation, and targeted accumulation [72,80].

**Conjugation with targeting ligands**

An important tactic for improving the therapeutic efficacy and tumor selectivity of drug delivery systems, including those that carry AMPs, is targeted ligand conjugation. To take advantage of receptor overexpression on tumor cells and encourage receptor-mediated internalization, ligands like folate, carbohydrates, peptides, aptamers, and antibodies can be covalently bonded to small-molecule medications or nanocarriers [81].

**(A)**  **Folate-Mediated Targeted Drug Delivery**

Due to the high expression of folate receptors (FRs) on many cancer cells and low expression on healthy tissues, folic acid (FA) is one of the most popular small-molecule targeting ligands. FA enters cells through receptor-mediated endocytosis via folate-binding membrane proteins and is essential for nucleotide biosynthesis and cell proliferation [81].

Folate-mediated tumor targeting uses two primary platforms:

- **Conjugates of Folate and Drugs**

Direct covalent attachment of folate to cytotoxic medications can enhance their selective uptake by cancer cells that overexpress FR.

II. **Nanoparticles Conjugated with Folate**

Through folate receptor-mediated pathways, nanocarriers functionalized with FA, such as liposomes, polymeric nanoparticles, and nanocapsules, exhibit improved internalization and increased tumor accumulation. One particular example is terpolymer-based nanocapsules functionalized with FA, in which the carboxyl groups of FA were covalently bound to amino groups in the DMAEMA monomer units after being activated using EDC/NHS chemistry. In contrast to nanocapsules containing only physically adsorbed FA, FT-IR demonstrated successful covalent attachment through the persistence of FA’s diagnostic carbonyl band after thorough washing [82].

**(B)**  **Carbohydrate-Mediated Targeted Drug Delivery**

Because they can interact with carbohydrate-binding receptors that are overexpressed in cancer cells, carbohydrates, which include monosaccharides, disaccharides, and polysaccharides, are ubiquitous biomolecules made of carbon, hydrogen, and oxygen that can act as ligands that target tumors [81]. There are two primary and principal techniques: drug-carbohydrate conjugates, in which drugs are directly linked to explicit saccharides to improve particular tumor absorption, and carbohydrate-conjugated nanoparticles, where carbohydrate-decorated nanocarriers improve tumor specificity and cellular absorption via receptor-mediated internalization [83].

**(C)**  **Targeting Mediated by Transferrin**

Peptide-functionalized nanocarriers and peptide-drug conjugates (PDCs) are potent instruments for tumor-targeted delivery. Transferrin receptor (TfR)-binding peptides are particularly interesting among peptide ligands because TfR is minimally expressed in normal tissues and highly overexpressed in many tumor types [84]. Conjugating antitumor drugs or nanocarriers with TfR-targeting peptides is expected to enhance particular tumor collection, encourage receptor-mediated endocytosis, lower off-target toxicity, and improve therapeutic effectiveness. Along with carbohydrates and folate, transferrin receptor-targeting peptides serve as effective ligands that substantially improve tumor selectivity, cellular absorption, and result in directed drug delivery systems [84].

### 5.3. Hydrogel and Microneedle-Based Systems for Localized Therapy

For the localized and minimally invasive delivery of therapeutic agents, such as peptides, small molecules, and biologics, microneedle (MN) technologies have shown great promise. These systems are able to deliver drugs to the local microcirculation by circumventing the stratum corneum barrier and enabling drugs to enter the microcirculation, resulting in targeted and controlled delivery with reduced systemic exposure. Many MN systems have shown significant potential in transdermal and localized therapy, and, in particular, hydrogel-based and microneedles, which respond to stimuli [85,86].

**(A)**  **Hydrogel microneedles**

Cross-linked hydrophilic polymer networks that can swell when they come into contact with interstitial fluid make up hydrogel-forming microneedles. These microneedles take up the surrounding biological fluids, soften or swell to form a channel in which therapeutic agents can be released gradually and continuously into the microcirculation upon insertion into the skin. Hydrogel MNs can be used to deliver drugs transdermally, vaccinate, and for other localized therapies due to biocompatibility and controllable release properties [87].

**(B)**  **Stimulus-Responsive Microneedles**

Micro-responsive needles are responsive to outside forces or internal physiological indications. Micelles fabricated utilizing smart biomaterials have enabled the delivery of drugs in precise and programmable quantities and locations. To be more precise and particular, activated microneedles are sources of on-demand, controlled delivery of drugs in a physical manner, for instance, light, ultrasound, or electrical fields. The systems improve the non-invasive versatility in conditions of therapeutic reactions since they allow clinicians or patients to alter the dosing parameters after utilizing the systems. Considering this illustration under light, a hydrogel micro needle that is light sensitive can be designed to entrap a drug like ibuprofen and be released under the influence of time and light to obtain the capability to deliver the drug to a local location, minimizing side effects [88]. The internally activated microneedle (MN) systems are activated by endogenous physiological signals to permit autonomous and site-specific drug delivery, with particularly compelling. intriguing, and striking examples of glucose-responsive microneedles to deliver insulin adaptively, pH-responsive microneedles to treat infections and heal wounds, and enzyme-responsive systems that permit flexible release profiles in diseased tissues. By integrating drug delivery with real-time biological conditions, these designs boost safety and effectiveness, while hydrogel-based and stimulus-responsive microneedle systems offer flexible, minimally invasive solutions for localized drug delivery in transdermal therapy, immunization, metabolic regulation, and wound care due to their swelling capability, environmental responsiveness, and controlled release behavior [89].

## 6. Preclinical and Clinical Evaluation

### 6.1. Summaries of In Vitro and In Vivo Studies Showing Tumor Regression

Many anticancer and oncolytic peptides– some of which are natural AMP-derived ACPs– have been recorded to show notable in vitro and in vivo antitumor activity [28]. These investigations demonstrate their ability to cause immunogenic cell death, alter the tumor microenvironment, and encourage long-term tumor regression. LTX-315 is a short cationic peptide that has been chemically altered and is derived from bovine lactoferrin. It has strong immunomodulatory and oncolytic effects. Its ability to eradicate tumors and create protective antitumor immunity has been shown in numerous studies.

**(A)**  **In Vitro Antitumor Mechanisms**

LTX-315 causes cancer cells to exhibit the traditional signs of immunogenic cell death (ICD), such as surface exposure of calreticulin, extracellular ATP release, nuclear exodus of HMGB1, and type-I interferon response activation. Together, these effects promote antitumor immunity and antigen presentation [90]. In rodent cancer models, LTX-315 has demonstrated significant in vivo efficacy. B16F10 melanoma (mice): Intratumoral injection caused the majority of the animals’ tumors to completely recede, and the cured animals were resistant to tumor re-challenge, indicating the development of adaptive immunity [91]. Mice with MCA205 sarcoma: Treatment reduced regulatory T cells and increased cytotoxic CD8^+^ T cells, which reprogrammed the tumor microenvironment and caused established tumors to regress. Intratumoral LTX-315 eradicated established tumors and boosted systemic, tumor-specific immunity in rats with rTMSC fibrosarcoma [92]. Further In Vivo Results in Mice with MCA205 Fibrosarcoma: LTX-315 mimicked the effects of mitoxantrone by causing transient hemorrhagic necrosis, massive HMGB1 release, caspase-3 activation, and T cell and macrophage infiltration [93]. When LTX-315 was injected into a single lesion in a multifocal tumor model (one intraperitoneal and two subcutaneous lesions), all tumor sites were eliminated, and a long-lasting remission was achieved, demonstrating strong systemic immunity [92]. The anti-proliferative activity of TNFα-encoding dendriplexes against several cancer cell lines (A431, B16-F10, T98G) was increased by conjugating lactoferrin (LF) and lactoferricin B (LFC) to DAB dendrimers. The enhanced cytotoxicity was ascribed to the enhanced transfection efficiency of dendriplexes modified with LF and LFC [94].

**(B)**  **Tumor Regression in Vivo**

Intravenous administration of DAB-LF and DAB-LFC dendriplexes produced potent tumoricidal effects, with A431 tumors showing 60% total tumor disappearance compared to 40% in the control dendriplex category. In the B16-F10 melanoma model, DAB-LF induced 20% tumor regression and 40% disappearance, while DAB-LFC attained 20% regression and 50% disappearance, outperforming the control (20% disappearance, 40% regression). In A431-bearing mice, all treated groups exhibited a 22-day extension in survival, whereas in B16-F10 models, 80% of the DAB-LFC-treated mice survived for 24 days [94]. Highly selective antitumor activity has been shown by the peptide R-DIM-P-LF11-334 [94].

**(C)**  **In vitro Cytotoxicity:**

Minimal toxicity toward human dermal fibroblasts, NHDF; Potent cytotoxicity toward human melanoma cell lines A375 and MUG Mel1 with LC^2^_0_ ≈ 10 μm. Melanoma cells are about 20 times more selective than normal cells [95]. In an A375 melanoma xenograft model, treatment reduced the tumor area by about 85% when compared to controls. Ki-67 immunohistochemistry verified a sharp decline in cell proliferation; tumors showed significant loss of proliferating cells and extensive fibrosis. R-DIM-P-LF11-334 caused tumor shrinkage through non-necrotic mechanisms rather than inducing necrosis, in contrast to LTX-315 and pleurocidin-family peptides [95]. Together, these studies demonstrate that antitumor peptides, such as R-DIM-P-LF11-334, lactoferricin-modified nanocarriers, and LTX-315, exhibit potent anticancer activity both in vitro and in vivo via mechanisms like immunogenic cell death, remodeling of the tumor microenvironment, selective cytotoxicity, and induction of systemic antitumor immunity. While these inquiries showed notable potential as anticancer agents, the clinical applications of ACPs are still challenging in view of issues such as hemolytic activity, off-target toxicity, poor serum stability, pharmacokinetic variability, and the incapacity to consistently achieve ideal tumor selectivity in vivo [96]. Based on the gold standard framework, the document demonstrates that LTX-315 meets all of the criteria for the legitimate induction of ICD. In the conclusion of the guidelines, it states that LTX-315 appears to qualify as an authentic ICD inducer and activates the coordinated emission of CALR, ATP, and HMGB1 [97]. In contrast, other agents, which were previously thought to activate ICD, such as cisplatin, do not necessarily qualify as genuine ICD inducers because they are unable to release all the individual immunogenic cell death markers. This suggests that LTX-315 is a more effective and comprehensive ICD inducer compared to cisplatin and potentially other therapies, as it is able to trigger the full complement of DAMP signals required for true immunogenic cell death [98]. Yet, numerous ACPs exhibit potent anticancer activity in the lab against cancer cell lines and show promising activity towards tumor-bearing animals, and these observations are not predictive of their activity in patients. However, successful clinical translation is frequently hindered by systemic toxicity, hemolytic activity, less ideal pharmacokinetics, proteolytic instability, immunological interactions, poor penetration into tumors, and differential tumor microenvironments. So, caution must be exercised when interpreting in vitro and in vivo preclinical data and not equating them to lead predictors of clinical success [19].

### 6.2. Clinical Translation and Current Status of Anticancer Peptide Clinical Trials

With several peptide medications approved for clinical use and numerous others moving through clinical trial pipelines, peptide-based therapeutics have made tremendous strides in cancer research. The therapeutic potential and increasing interest in anticancer peptides in research are reflected in the current development landscape [16,99].

**(A)**  **Anticancer peptides that have been approved**

Among the anticancer therapies established on peptides, only a few have so far received any regulatory agency consent, including Buserelin, Tebentafusp, Plitidepsin, Triptorelin, and Dactinomycin. Unlike classical membranolytic ACPs, which have anticancer effects chiefly by disrupting the membranes of cancer cells, plitidepsin chiefly acts on intracellular molecular processes. Plitidepsin binds to the eukaryotic elongation factor 1 alpha 2 (eEF1A2) protein, which is overexpressed in various sorts of cancers, producing oxidative stress, interruption of protein homeostasis, and activation of the apoptosis-related signaling pathway. In light of these outcomes, nonetheless, the term “mechanically distinct” peptide-based anticancer agent is a better description to employ for plitidepsin than the one conventionally utilized, “membrane-active ACP”. Triptorelin for the palliative treatment of advanced prostate cancer; Dactinomycin for a wide range of malignancies [16].

**(B)**  **Active Clinical Trials for Anticancer Peptides**

According to the document, 400–600 peptides are now undergoing preclinical evaluation, and over 150 peptide medications are in clinical development. Several anticancer peptides are under inquiry, incorporating thirteen clinically examined peptides: Tigapotide, SF1126, ATN-161, Teverelix, IRL-1620, Nelipepimut-S, Iseganan, G17DT, Canfosfamide, PM02734, CTCE-0214, Darinaparsin, and Labradimil; as well as eleven supplementary peptide prospects in study, specifically Ozarelix, Soblidotin, LTX-315, Balixafortide, VEGFR2–169, Bombesin, Valspodar, TAK-448, Dolastatin 10, Zoptarelin doxorubicin, and Blemomycin A6, which are in distinct stages of clinical evaluation [99].

**(C)**  **Recently Completed and Ongoing Clinical Trials**

A total of 22 post-radical prostatectomy patients participated in a phase I/II trial, which showed excellent safety and no grade 3 treatment-related adverse events [100]. NY-ESO-1 ISCOMATRIX Vaccine for Melanoma, Phase II trial, 46 patients with Stage II-IV melanoma, induced a strong and long-lasting CD4^+^ T-cell response [101]. HORIZON Study: Dexamethasone + Melphalan Fluperamide (Melflufen), Phase III trial, recruitment is still ongoing, assessing effectiveness in multiple myeloma that has relapsed or is refractory [102].

### 6.3. Pharmacokinetics, Biodistribution, and Toxicity Considerations

The pharmacokinetic behaviors of Bi-based nanomaterials are best described by one- or two-compartment models, with PEGylated Bi nanoparticles showing a clearance half-life of 3–5 h, ultrasmall Bi_2_Se_3_ nanodots coated with BSA showing a distribution half-life of 0.16 h and an eradication half-life of 1.49 h, and hollow (BiO)_2_CO_3_ nanotubes presenting a distribution half-life of 27.6 min and a removal half-life of 26.99 h, demonstrating that size and surface chemistry strongly affect circulation behavior [103]. Minimal cytotoxicity in vitro; No notable physiological abnormalities or tissue damage in animal models; Effective removal of dissolved Bi^3+^ ions through metallothionein pathways. All of the data points to Bi-based nanomaterials’ potential for translation and biocompatibility [103].

## 7. Challenges and Limitations

### 7.1. Proteolytic Degradation and Stability

**(A)**  **Instability of Nucleic Acid and Peptide Nanostructures**

One of the important and significant barriers to the clinical success of peptide-based and self-assembling biomaterials is stability, as nucleic acid and peptide nanostructures are inherently unstable and volatile in vivo due to fast enzymatic decline. Nucleic acid-based self-assembling nanostructures are easily degraded by RNases, leading to poor systemic persistence, and RNA nanoparticles lacking covalent linkages tend to dissociate upon extreme dilution following systemic administration [104].

**(B)**  **Proteolytic Susceptibility of Self-Assembling Peptides (SAPs)**

Before SAPs reach their in vivo destination, proteolytic enzymes may break them down, decreasing their therapeutic efficacy. Adding D-amino acids is a common stabilization technique because D-peptides show prolonged circulation while L-peptides are broken down quickly [104].

**(C)**  **Enzymatic Instability of AMPs**

AMPs usually suffer from speedy proteolysis, with a lifespan of approximately 30 min due to deterioration by serum proteases, intestinal fluids, and liver/kidney clearance [105]. To address this instability, various approaches are utilized, comprising cyclization (head-to-tail, head-to-side chain, and side-to-side chain), terminal changes such as N-acylation, C-amidation, N-pyroglutamate formation, PEGylation, and sialylation, replacement with non-natural amino acids (D-amino acids, β-, γ-amino acids, and N-methyl-α-amino acids), pseudopeptide formation through N-alkylation, carbonyl or NH-group replacement, co-administration with enzyme inhibitors, and encapsulation in liposomes, nanoparticles, or polymer carriers [105].

### 7.2. Techniques for Stability Assessment

HPLC-MS quantification after incubation in human plasma or serum at 37 °C. Testing for stability in gastrointestinal fluids, membrane vesicles, and liver/kidney homogenates. Peptide Cutter and other in silico cleavage prediction tools [106].

### 7.3. Non-Selective Toxicity at Higher Doses

**(A)**  **Dose-Toxicity Relationships**

The monotonic toxicity assumption is supported by the consistent correlation between higher doses and increased toxicity. Conversely, efficacy does not always rise with dosage, suggesting that dose-escalation techniques might suggest dosages that are needlessly harmful and ineffective [107]. Even though AMPs are typically less toxic than conventional antibiotics, a number of risks still exist: Particularly at high concentrations, some AMPs cause host cell cytotoxicity or damage host membranes [108]. AMPs may exhibit dose-dependent, non-selective toxicity in anticancer applications, which would prevent safe clinical translation [109,110]. Despite the encouraging anticancer activity of numerous ACPs, their therapeutic selectivity remains an important and significant difficulty for clinical translation. Several ACPs exhibit hemolytic activity, off-target cytotoxicity toward normal mammalian cells, dose-limiting toxicity, and poor serum stability, arising in narrow therapeutic windows. Furthermore, the degree of selectivity varies significantly depending on peptide sequence, physicochemical characteristics, cancer type, membrane composition, and delivery approach. Therefore, although ACPs demonstrate notable potential as anticancer therapeutics, their safety and selectivity profiles need careful optimization before pervasive and far-reaching clinical application [111].

**(B)**  **Strategies to Reduce Toxicity**

Toxicity can be reduced by nano formulations: Melittin encapsulation in Poloxamer 188 preserved antitumor activity while reducing systemic toxicity and inflammatory allergic reactions in mice [112]. Despite their potential, it is still difficult to achieve truly selective anticancer peptide activity [49,113].

### 7.4. High Production Costs and Formulation Challenges

Manufacturing peptide-based therapeutics requires complex biophysical optimization:

**(A)**  **Solubility and Concentration Constraints**

High concentrations can cause aggregation, viscosity increases, and structural instability; peptides and proteins frequently have solubility restrictions that limit high-concentration formulation [114].

**(B)**  **Scale-Up and Manufacturing Complexity**

Peptide conformation may become unstable during large-scale production, which could impact yield and bioactivity [115].

**(C)**  **Regulatory and Delivery Barriers**

There are major safety and regulatory obstacles for novel delivery systems, which raise costs and development time [116].

### 7.5. Resistance Mechanisms and Immunogenic Responses

One way to classify resistance is as follows: Loss of antigenicity (lower immune cell recognition), Immunogenicity loss (inability to induce immune activation). The development of a microenvironment that suppresses immunity [117].

Despite encouraging preclinical results, the successful clinical translation of ACP-based therapeutics remains limited by various biological, pharmaceutical, and regulatory hurdles [117]. Major difficulties include systemic toxicity, hemolytic activity, quick enzymatic decline, poor serum stability, limited bioavailability, short circulation half-life, insufficient tumor specificity, immunogenicity concerns, and irregular pharmacokinetic behavior. In addition, large-scale peptide production, formulation stability, production costs, and regulatory authorization pathways persist as significant barriers for commercialization and pervasive and far-reaching clinical implementation [118].

Several emerging plans are being examined to overcome these limitations, including peptide cyclization, incorporation of non-natural amino acids, PEGylation, peptide conjugation techniques, nanocarrier-based delivery systems, and tumor-targeted formulations. Combination therapies and advanced delivery technologies may further improve therapeutic selectivity, stability, and clinical effectiveness. Nevertheless, substantial translational and clinical validation is still required before ACPs can achieve broader clinical application in oncology [119].

### 7.6. Adaptive and Primary Immune Resistance

Adaptive immune resistance, which frequently involves PD-1/PD-L1 pathways, happens when tumors change their phenotype in reaction to T-cell attack [120,121]. Tumor features that hinder immune recognition are the root cause of primary immune resistance [120].

### 7.7. Interferon Signaling Functions

Depending on the tumor context, interferon pathways can either promote immunosuppressive signaling or improve antigen presentation and immune activation [122]. Designing successful immunotherapies requires an understanding of these mechanisms through responder vs. non-responder comparisons [120].

## 8. Future Perspectives

Hybrid peptides and peptide drug conjugates represent thrilling methods to increase the specificity, stability, and effectiveness of cancer treatments. Structural modifications such as cyclization, replacement of amino acids, N-methylation, and PEGylation provide increased stability, increased membrane permeability, and half-life for peptides. PDCs include the application of targeting peptides (such as RGD and GnRH) linked with cytotoxic agents (such as doxorubicin and gemcitabine). Incorporation of nanotechnology into peptides through techniques involving the application of nanoparticles made from metals such as gold/silver, solid lipid nanoparticles, and even chitosan increases the stability, delivery, and reduces the toxicity of peptides. Nonetheless, peptide drugs face obstacles in conditions of poor stability triggered by enzymatic breakdown, reduced half-lives, toxicity, immunogenicity, difficulty in delivery, and high costs. Future inquiry is increasingly focused on advanced AI architecture, specifically Large Language Models (LLMs), Graph Neural Networks (GNNs), and structure-guided design. These instruments are being used to navigate the immense sequence space of peptides [123].

## 9. Conclusions

Developments in the engineering and delivery of ACPs, comprising peptides derived from naturally occurring AMPs, have supplied innovative and directed methods for cancer therapy. Although there is significant preclinical evidence, there are numerous barriers to the clinical application of these agents, including instability, low bioavailability, toxicity, complex and intricate synthesis operations, and immunological problems. Importantly, promising in vitro cytotoxicity and tumor regression in animal models do not invariably align with successful clinical results, highlighting the need for exhaustive and stringent translational and clinical evaluation of ACP-based therapeutics.

There are multiple solutions being developed for these challenges, comprising nanoparticle delivery, hybrid peptide methods, and chemical alterations, among others. As a result, with the proper large-scale production processes, rules, and preclinical evaluation procedures, overall, ACPs represent promising multifunctional therapeutic candidates for precision oncology; nonetheless, crucial and vital translational hurdles, including systemic toxicity, instability, immunogenicity, production intricacy, limited tumor selectivity, and regulatory problems, must still be overcome before broader clinical implementation can be accomplished.

## Figures and Tables

**Table 1:** AMPs and ACPs can be divided into five broad classes: α-helical, β-sheet, αβ-mixed, and non-αβ/extended peptides, and cyclic peptides.

Structural Class	Description/Mechanism	Examples	References
α-helical	These powerful short amphipathic (cationic) sequences adopt helical conformations upon contact with membranes. The anticancer activity of α-helical ACPs is strongly linked to their hydrophobicity, orientation, and ability to destabilize the packing of lipids in cancer cell membranes, leading to necrosis through membrane insertion.	Magainin II, Aurein 1, L-K6, LL-37, Melittin	[14,15]
β-sheet	β-sheet peptides can develop more rigidity from two or more disulfide bonds. These peptides are common in animals and plants. The represented ACPs bovine lactoferrin (LfcinB) and human neutrophil peptide (HNP-1) are examples of membrane-disruptive activity due to their ability to form pores as described with SVS-1 in lung, epidermal, and breast cancer cells.	LfcinB, HNP-1	[16]
αβ-mixed	αβ-mixed peptides feature both helical and sheet domains. The structural flexibility afforded by combined helical and β-sheet architecture facilitates dynamic interactions with cancer cell membranes.	Human β-defensin-3	[17]
Non-αβ/Extended	Non-αβ or extended/coil peptides are also not represented by regular secondary structure and are enriched with the residues tryptophan, proline, and glycine. These peptides can exist in these flexible conformations to insert deep into lipid bilayers.	Indolicidin, Alloferon, PR-39	[17,18]
Cyclic peptides	Cyclic peptides often display increased stability compared to their linear counterparts due to head-to-tail cyclization or disulfide-bonded loops.	Diffusa Cytide 1-3, H-10	[19]

**Table 2:** Source diversity of AMPs and ACPs.

Source Category	AMPs	ACPs	References
Plants Derived	Examples are thionins, defensins, hevein-like peptides, knottins, α-hairpinins, lipid transfer proteins, snakins, and non-cysteine-rich peptides; Thi2.1, Mj-AMP2, petunia defensins, PmAMP1, SN-1.	Grifficyclocin B (*Goniothalamus* spp.); acts via membrane disruption and apoptosis induction.	[25,26]
Animals Derived	Found in mammals, reptiles, amphibians, fish, invertebrates; examples: LL-37, indolicidin, protegrins, HNP-1-4, HBD-1-4, histatins, dermcidin.	Induction includes jaspamide, dolastatin 10, melittin, gomesin, pardaxin, magainin 2, crotamine, LL-37; mechanisms include membrane disruption, apoptosis, and anti-angiogenesis.	[27,28]
Microorganisms Derived	Synthesized by non-ribosomal peptide synthetases (NRPSs); examples: nisin (Lactococcus lactis), mersacidin (Bacillus sp), PAF (Penicillium chrysogenum).	Includes bacteriocins and other peptides causing membrane damage, oxidative stress, and apoptosis.	[29,30]
Synthetic and Engineered AMPs	Chemically synthesized and designed AMPs such as pexiganan, omiganan, LTX-109, brilacidin; recombinant production of diverse and varied AMPs.	Modified ACPs with enhanced stability and selectivity via D-amino acids, cyclization, targeting (RGD, TAT), nanoparticles; example: LTX-315 (clinical trials).	[25,27,29,30]

**Table 3:** Four key physicochemical parameters: Net charge, Hydrophobicity, Amphipathicity, and Peptide length, govern AMPs and ACPs membrane interactions and overall bioactivity.

Parameter	AMPs	ACPs
Net Charge	These peptides interact with negatively charged bacterial membranes through electrostatic forces. Increased positive charge increases membrane disruption but may increase toxicity for eukaryotic cells due to excessive charge [47]. Example: Bac2A shows enhanced activity due to higher charge [48].	ACPs utilize positively charged amino acid residues (Arginine, Lysine) to target cancer cells having Membranes containing phosphatidylserine. Arginine to Lysine replacement may help retain activity and minimize toxicity [49].
Hydrophobicity	Hydrophilic residues assist membrane targeting, whereas hydrophobic residues (Tryptophan, Phenylalanine) improve activity but reduce selectivity and toxicity [50]. QSAR shows hydrophobic patch size is critical (max S5 > 2) [51].	Increased hydrophobicity stabilizes helical structure and enhances tumor penetration and cytotoxicity, but excessive hydrophobicity leads to hemolytic toxicity [52].
Amphipathicity	The amphipathicity of helix/sheet enables proper interaction and disruption of the membrane. Excessive rigidity can result in toxicity. CD results indicate that the active peptides assume a structured conformation on membrane binding [47,53].	Membrane disruption by ACPs occurs through their amphipathicity. Proper hydrophobic and hydrophilic balance is important for selectivity [54].
Peptide Length	Small peptides (10–20 amino acid residues) are active but can affect selectivity and potency. Length of the peptide is essential to maintain therapeutic effectiveness. For instance, Bac2A and 12-mer derivatives exhibit satisfactory activity [48].	Reduced ACPs maintaining essential domains boost efficiency, tissue permeability, economy, and pharmacokinetic attributes [17]. Enhanced hydrophobicity (for example, V13KL variants) results in increased activity; however, high hydrophobicity decreases efficiency and causes hemolytic reactions [55,56].

## Data Availability

No new data were created or analyzed in this study. Data sharing is not applicable to this article.

## Abbreviations

AMPs	Antimicrobial Peptides
ACPs	Anticancer Peptides
ICD	Immunogenic Cell Death
VEGF	Vascular Endothelial Growth Factor
SAR	Structure-Activity Relationship
